# Effect of Colchicine in reducing MMP-9, NOX2, and TGF- β1 after myocardial infarction

**DOI:** 10.1186/s12872-023-03464-9

**Published:** 2023-09-11

**Authors:** Suryono Suryono, Mohammad Saifur Rohman, Edi Widjajanto, Seskoati Prayitnaningsih, Titin Andri Wihastuti, Yudi Her Oktaviono

**Affiliations:** 1 Doctoral Program of Medical Science, Brawijaya University, Malang, East Java, Indonesia; 2 Department of Cardiology and Vascular Medicine, Faculty of Medicine, Jember University, Jember, East Java, Indonesia; 3 Department of Cardiology and Vascular Medicine, Faculty of Medicine, Brawijaya University, Malang, East Java, Indonesia; 4 Brawijaya Cardiovascular Research Centre, Brawijaya University, Malang, East Java, Indonesia; 5 Department of Clinical Pathology, Faculty of Medicine, Brawijaya University, Malang, East Java, Indonesia; 6 Department of Ophthalmology, Faculty of Medicine, Brawijaya University, Malang, East Java, Indonesia; 7 Department of Biomedical, Nursing Science, Faculty of Medicine, Brawijaya University, Malang, East Java Indonesia; 8 Department of Cardiology and Vascular Medicine, Faculty of Medicine, Airlangga University, Surabaya, Indonesia

**Keywords:** Colchicine, MMP-9, NOX2, PCI, STEMI, TGF-β1

## Abstract

**Background:**

According to WHO 2020, CAD is the second leading cause of death in Indonesia with death cases reaching 259,297 or 15.33% of total deaths. Unfortunately, most of the patients of CAD in Indonesia did not match the golden period or decline to be treated with Percutaneous Coronary Intervention (PCI). Based on the recent study, there were increases in MMP-9, NOX2, and TGF-β1 in STEMI patients which contribute to cardiac remodeling. Moreover, there is controversy regarding the benefit of late PCI (12-48 hours after onset of STEMI) in stable patients. Lately, colchicine is widely used in cardiovascular disease. This study was conducted to explore the effect of colchicine to reduce MMP- 9, NOX2, and TGF-β1 levels after myocardial infarction in stable patients.

**Method:**

In this clinical trial study, we assessed 129 STEMI patients, about 102 patients who met inclusion criteria were randomized into four groups. Around 25 patients received late PCI (12–48 h after the onset of chest pain), optimal medical treatment (OMT) for STEMI, and colchicine; 24 patients received late PCI and OMT; 22 patients didn’t get the revascularization (No Revas), OMT, and colchicine; and 31 patients received No Revas and OMT only. The laboratory test for MMP-9, NOX2, and TGF-β1 were tested in Day-1 and Day-5. The data were analyzed using Mann-Whitney.

**Results:**

A total of 102 patients with mean age of 56 ± 9.9, were assigned into four groups. The data analysis showed significant results within No Revas + OMT + Colchicine group versus No Revas + OMT + Placebo in MMP-9 (Day-1: p = 0.001; Day-5: p = 0.022), NOX2 (Day-1: p = 0.02; Day-5: p = 0.026), and TGF-β1 (Day-1: p = 0.00; Day-5: p = 0.00) with the less three markers in OMT + Colchicine group than OMT + Placebo group. There were no significant differences within the late PCI + OMT + colchicine group and PCI + OMT + Placebo group.

**Conclusions:**

Colchicine could significantly reduce MMP-9, NOX2, and TGF-β1 levels in stable STEMI patients. So that, colchicine could be a potential agent in STEMI patients and prevent cardiac remodeling events.

## Introduction

Coronary artery disease (CAD) is a disease caused by an inadequate supply of blood and oxygen to the myocardium. This condition made the oxygen demand higher than the oxygen supply to the myocardium, which occurred from the complete or partial occlusion of the coronary arteries lumen due to atherosclerotic plaque [1]. There are some conditions due to CAD, such as myocardial infarction (MI), stable angina, unstable angina, and also sudden death [2]. CAD is very common in both developed and developing countries. Brown et al. estimated that CAD represented 2.2% of the overall global burden of disease and 32.7% of cardiovascular diseases [3]. According to the latest WHO data published in 2020, CAD is the second leading cause of death in Indonesia with death cases reaching 259,297 or 15.33% of total deaths. The age-adjusted death rate is 125.99 per 100,000 of the population in Indonesia is ranked 70 in the world [4]. If the coronary arteries are seriously blocked, blood flow may not be adequate for any increased demand, such as that of exercise or an emotional upset.

When there is complete occlusion which makes myocardial cells necrosis, it will become myocardial infarction (MI) and might show the ST-elevation myocardial infarction (STEMI) pattern in ECG [5]. Percutaneous coronary intervention (PCI) still becomes an important treatment for STEMI. PCI could open up the infarct-related artery and prevent re-occlusion. However, in the real world, after being transferred to the PCI center, many patients have missed the optimal PCI time or even refused the PCI, especially in developing countries including Indonesia [6, 7]. This condition is caused by many reasons such as chest pain denial, poor access to the PCI center, poor economic condition, and refusal to be done by PCI procedure. However, there are controversies regarding the benefit of late PCI (12-48 hours after onset of STEMI) in stable patients, since the current publications showed different results [8].

MI could develop into cardiac remodeling through the cellular, molecular, and proteomic process which causes ventricular hypertrophy. This condition promotes myocardial wall change, contractility impairment, and disturbance of systolic and diastolic function, so it could lead to heart failure [9]. Several biomarkers such as Matrix Metalloproteinases-9 (MMP-9), nicotinamide adenine dinucleotide phosphate (NADPH) oxidases or NOX2, and TGF-β1 are potent markers to investigate the cardiac remodeling process. Previous studies reported that those markers were elevated in the ischemic and penumbra area of the heart after MI [10]. A recent study showed that the NOX2 level elevated in post-MI areas than in the healthy areas of the heart [11]. The increment of NOX2 contributes to ventricular remodeling and heart failure in MI through ROS activation [12]. The study conducted by Lindsey in 2018 showed that MMP-9 which regulates inflammation by recruiting neutrophils and macrophages, is elevated in MI patients with high mortality and leads to CAD condition [13]. MMP-9 may trigger the activation of Transforming Growth Factor Beta 1 (TGF-β1). At the site of infarction, TGF-β1 induced the differentiation of interstitial fibroblasts into fibroblasts that contain huge collections of actin microfilaments that may promote ventricle remodeling [14].

In order to prevent heart failure due to cardiac remodeling, it is important to regulate markers that are elevated in MI conditions that could lead to cardiac remodeling. Along with the development of research, colchicine is widely used in the treatment of cardiovascular diseases. Colchicine is a potent agent which could exhibit some anti-inflammatory actions by inhibiting neutrophil chemotaxis, inflammasome network, and pro-inflammatory cytokines [15]. Study by Suryono et al., which tested the inhibitory effect of colchicine targeting MMP-9, NOX2, and TGF-β1, showed that colchicine has a good docking score on those three molecules and could specifically bind to the active sites of those molecules, and also colchicine has high stability when it binds to MMP-9, NOX2, and TGF-β1 through the molecular docking and MD simulation analysis [16]. On the other hand, there is a study that shows that colchicine has a correlation with the decrease of MMP-9 and NOX2 [17]. We have a hypothesis that suppressing MMP-9, NOX2, and TGF-β1 which correlated to cardiac remodeling by colchicine, may improve the outcome in MI patients. Therefore, we conduct a clinical-trial study to demonstrate the potency of colchicine and late PCI to regulate the progression of MMP-9, NOX2, and TGF-β1 in stable MI patients who are done by late PCI and Optimal Medical Treatment (OMT) only.

## Method

### Clinical trial registration

This study was registered in clinicaltrials.gov with the registration code: NCT05709509 (02/02/2023) - Effect of Colchicine on MMP-9, NOX2, and TGF-β1 in Myocardial Infarct.

### Study population

**Fig. 1:**
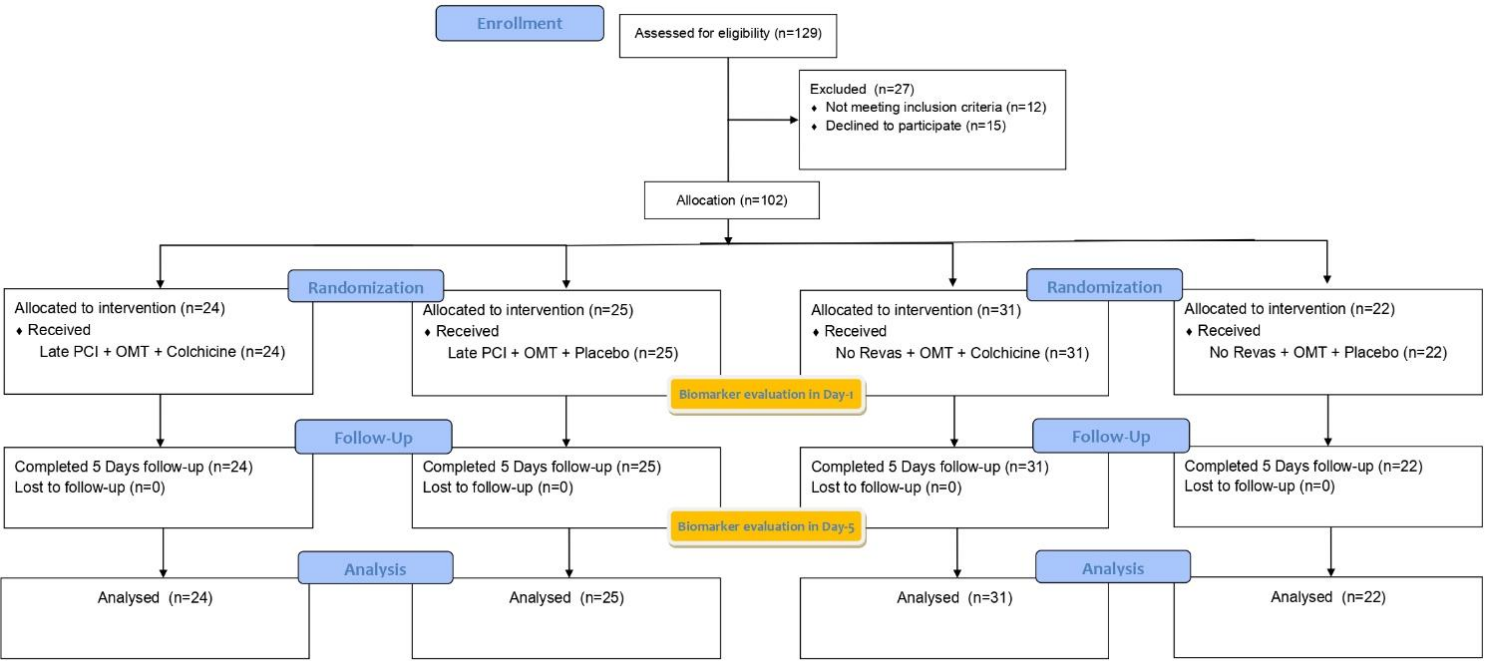
CONSORT Flow Chart. PCI: Percutaneous Coronary Intervention; CONSORT: Consolidated Standards of Reporting Trials

We included all consecutive patients referred to 3 Hospitals in East Java, Indonesia: Soebandi, Saiful Anwar, and Iskak Hospitals from June 2022 until December 2022. Treatment decisions were made by the cardiologist and the patient in consultation, and the procedure and location of the stent placement were entirely by the interventionist of the cardiologist. Patients who met the inclusion criteria were randomized into four groups using a 1:1 allocation scheme based on a computer-generated randomization algorithm. We included 102 patients from 129 patients (Fig. 1).

### Criteria for inclusion and exclusion

#### Inclusion criteria

The patient was presented with STEMI between 12 and 48 h from the onset of chest pain, 40–70 years old, and received the informed concern.

#### Exclusion criteria

Main exclusion criteria were age < 40 or > 70 years; comorbid disease such as such as infection, inflammation, malignancy, severe renal failure (EGFR < 30), a history of hepatic cirrhosis, acute exacerbation of hepatitis, or severe liver disease; alcoholic patient, cardiac arrest; ventricular fibrillation or cardiogenic shock; unstable hemodynamic; and refuse to received coronary intervention.

### Blinding and randomization

In our study, we randomly assigned participants to receive either colchicine or a placebo for 5 days based on their informed consent agreement (patients who agreed to late PCI will join the late PCI group, and those who refused the late PCI will join the No Revas group). Tablets for colchicine (manufactured by Pratapa Nirmala (Fahrenheit), Indonesia) and the placebo (prepared by the pharmacy department of Saiful Anwar General Hospital) had the exact same color, design, and packaging. The patients, doctor, and pharmacist who conducted the study’s evaluation were all unaware of the intervention’s assignment.

#### Treatment

All patients were treated with the optimal medical treatment for STEMI, including aspirin, P2Y12 inhibitor, statin, beta blocker, LMWH, and nitrate based on the patient’s condition. We randomized all patients into four groups. The first group consisted of patients who received late PCI with the loading of colchicine 1 mg 1 h before late PCI, and colchicine 0.5 mg 1 h after late PCI was done, and continued with colchicine 0.5 mg daily for 5 days. The second group received late PCI and optimal medical treatment for 5 days. The third group consisted of patients who got optimal medical treatment and received the loading of colchicine 1 mg 1 h before entering ICCU, colchicine 0.5 mg 1 h after that, and continued with colchicine 0.5 mg daily for 5 days. The fourth group are patients who get optimal medical treatment for 5 days without colchicine.

### Data extraction

During 24 h after late PCI (for late PCI group) and 24 h after ICCU admission (for No Revas group), the laboratory test of MMP-9, NOX2, and TGF-β1 was done for each four groups which measured by Enzyme-linked Immunosorbent Assays (ELISA) (BT Laboratory™) method using 3 cc blood vein sample from the patients in each group. The laboratory tests of MMP-9, NOX2, and TGF-β1 all performed in the Laboratory of Physiology, Animal Structure and Development, Molecular Biology Building (Biomol), Faculty of Mathematics and Natural Sciences, Brawijaya University, Indonesia. After preparing all the reagents needed for each marker, 40 µl blood sample for each marker and 10 µl antibody (anti-MMP-9, anti-NOX2, and anti- TGF-β1) were added in the plate for each marker, and then 50 µl streptavidin-HRP was mixed into each plate. After that 50 µl A substrate, 50 µl B substrate, and 50 µl stop solution were added in each plater. The absorbance was measured using 450 nm spectrofluorometry. About 5 days after treatment, the second laboratory test of MMP-9, NOX2, and TGF- β1 was done again for each four groups. The standard laboratory test including Hemoglobin, leukocyte, thrombocyte, blood urea nitrogen, creatinine serum, aspartate transaminase, alanine transaminase, and troponin were immediately tested for all of the patients.

### Endpoint

The primary endpoint event is the effect of colchicine in regulating MMP-9, NOX2, and TGF-β1 which are elevated in MI patient that contribute to cardiac remodeling, which evaluated from the amount of MMP-9, NOX2, and TGF-β1.

### Statistical analysis

Descriptive data such as age, gender, risk factors and comorbid, laboratory test, and also infarct locations are presented in numbers. For the analysis of numerical data, the normality test was performed using Kolmogorov Smirnov. Then the difference test between groups is carried out using the Mann-Whitney test. Data analysis in this study was assisted by the SPSS 26 for Windows program.

### Result

#### Baseline characteristics

Figure 1 showed the CONSORT flow chart of this research. About 129 patients were assessed for eligibility. Amongst them, 27 were excluded due to several reasons (12 patients did not meet the inclusion criteria: there was 1 patient with GI tract abnormality in the late PCI + OMT + colchicine group; there was 1 patient with GI tract abnormality, 1 patient with cardiogenic shock, 1 patient with renal impairment, and 1 patient with liver impairment in late PCI + OMT + Placebo group; there was 1 patient with GI tract abnormality and 1 patient with cardiogenic shock in No Revas + OMT + colchicine group; 1 patient with cardiogenic shock, 1 patient with renal impairment, 1 patient with liver impairment, and 2 patients were die in No Revas + OMT + Place group. Another 15 patients declined to participate). Around 102 patients were eligible and allocated to four types of intervention including Late PCI + OMT + Colchicine, Late PCI + OMT + Placebo, OMT + Colchicine, and OMT + Placebo. All subjects completed the study phase. The late PCI group consists of Late PCI + OMT + Colchicine group and Late PCI + OMT + Placebo group. Hence the No Revas group consists of No Revas OMT + Colchicine group and No Revas + OMT + Placebo group. The allocation of the subjects in Late PCI group or No Revas group is not randomized but depends on the subject’s authority. If the subjects choose to be treated with late PCI, the subjects were randomized into one of the Late PCI groups and vice versa.

Table 1 summarizes the baseline of a patient’s characteristics according to treatment modality and demographic data. The mean age was 56 years old (46–66), and 64.7% of the patients were male. In this study, 53% had a medical history of hypertension, 21.5% had diabetes, 14.7% had dyslipidemia, 61.7% are smokers and ex-smoker, and 52.9% have large infarcts. Based on the significant value, there was no correlation between the risk factor and the event of infarction in each group (*p* value > 0.05). There were no significant differences (*p* value > 0.05) in the type of infarct (large or small) in each group, so the type of infarct area did not affect the data in each group.

**Table 1:** Demographic Data

Variabel	Categories	Late PCI + Placebo (n = 24)	Late PCI + Colchicine (n = 25)	No Revas + Placebo (n = 31)	No Revas + Colchicine (n = 22)	Total (n = 102)	*P* *Value*
**Age**	-	53 ± 11.2	56 ± 8.8	59 ± 8.8	56 ± 10.7	56 ± 9.9	0.288
**Sex**	Male	18	14	18	16	66	
	Female	6	11	13	6	36	0.167
**Hypertension**	Yes	12	15	16	12	55	
	No	12	10	15	10	47	0.486
**Diabetes**	Yes	2	6	13	4	25	
	No	22	19	18	18	77	0.142
**Dyslipidemia**	Yes	3	5	5	2	15	
	No	21	20	26	20	87	0.482
**Smoking / Ex- Smoker**	Yes	17	13	17	16	63	
	No	7	12	14	6	39	0.181
**Type of** **Infract**	Large Infract	15	10	14	15	54	
							0.119
	Small Infarct	9	15	17	7	48	

Note: Large infarct: Anterior, Anteroseptal, Anterolateral, Anterior extensive

Small infarct: Inferior

the test used is Mann-Whitney test

**Fig. 2:**
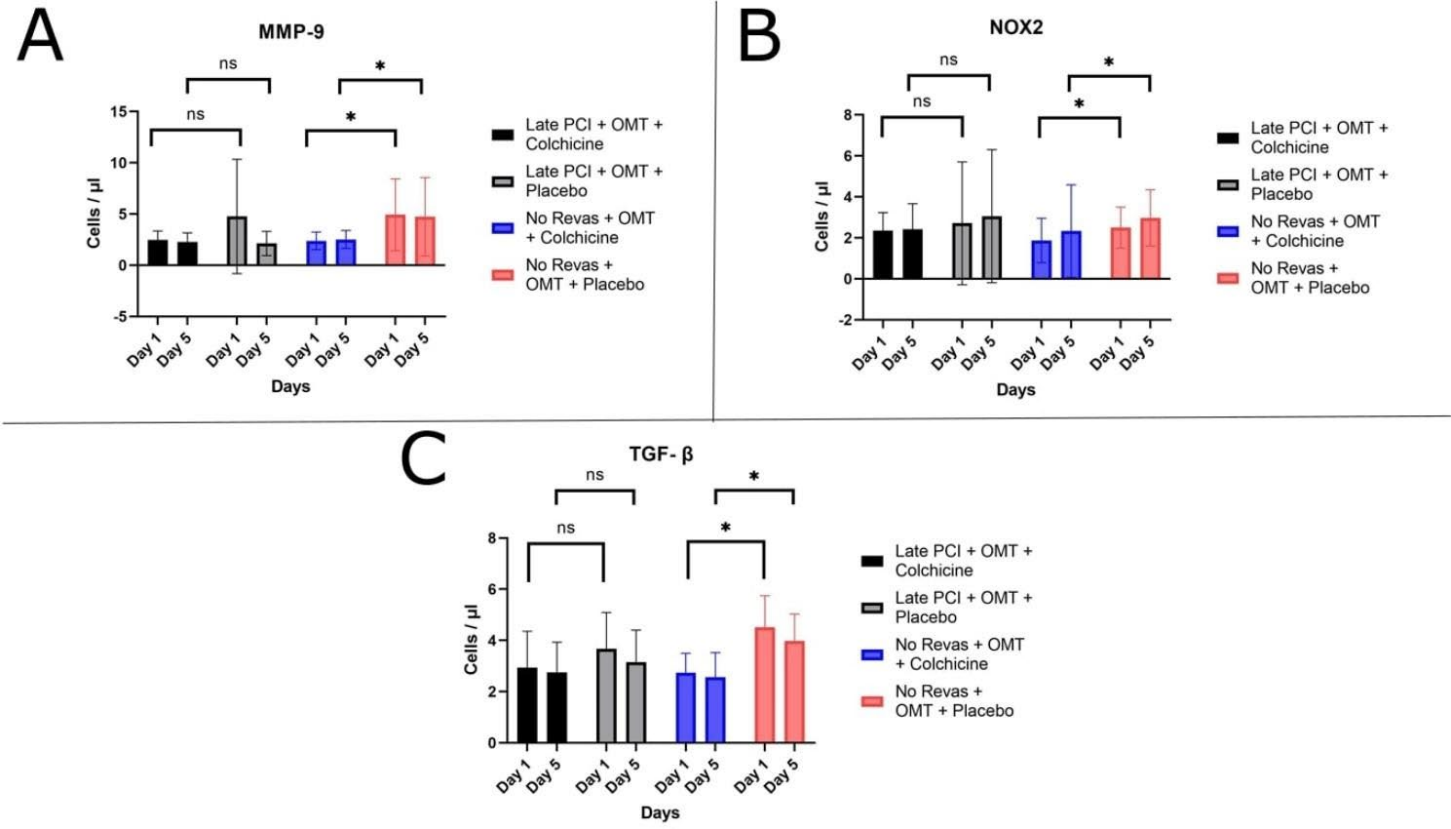
Analysis of biomarkers between each group during Day-1 and Day-5 (**A**) Analysis of MMP-9 levels; (**B**) Analysis of NOX2 levels; (**C**) Analysis of TGF-β levels *p = < 0.05; ns = not significant

Figure 2 showed the difference in MMP-9, NOX2, and TGF-β levels during Day-1 and Day-5. The exact number of mean ± SD from all biomarkers can be seen in Table 2. Based on the results, if we compare all the biomarkers on Day-1 versus Day-5 within the same biomarker, there are no significant differences between all data. In the late PCI group, in Late PCI + OMT + Colchicine versus Late PCI + OMT + Placebo group in the same day, there are no significant differences in MMP9 (Day-1: p = 0.59; Day-5: p = 0.93), NOX2 (Day-1: p = 0.78; Day-5: p = 0.14), and TGF-β (Day-1: p = 0.053; Day-5: p = 0.14). Nonetheless, there is no significant difference, the trends between all biomarkers revealed higher levels of biomarkers in Late PCI + OMT + Placebo than Late PCI + OMT + Colchicine group. However, we found a noticeable difference in the No Revas group which consists of No Revas + OMT + colchicine group and No Revas + OMT + Placebo group.

The trends in the No Revas group showed that all of the No Revas + OMT + Placebo groups had higher MMP-9, NOX2, and TGF-β levels than the No Revas + OMT + Colchicine group both in the Day-1 and Day-5. The analysis between each biomarker showed significant results within No Revas + OMT + Colchicine group versus No Revas + OMT + Placebo in MMP9 (Day-1: p = 0.001; Day-5: p = 0.022), NOX2 (Day-1: p = 0.02; Day-5: p = 0.026), and TGF-β (Day-1: p = 0.00; Day-5: p = 0.00). The exact number of mean ± SD from all biomarkers can be seen in Table 2.

**Table 2:** Summary of biomarkers analysis: MMP-9, NOX2, and TGF-β1

Biomarkers	Categories	Late PCI + Colchicine (mean ± SD)	Late PCI + Placebo (mean ± SD)	No Revas + Colchicine (mean ± SD)	No Revas + Placebo (mean ± SD)
MMP-9	Day-1	2.46 ± 0.88	4.77 ± 5.58	2.38 ± 0.85*	4.93 ± 3.50*
	Day-5	2.27 ± 0.88	2.13 ± 1.18	2.51 ± 0.88*	4.73 ± 3.82*
NOX2	Day-1	2.36 ± 0.87	2.71 ± 2.99	1.87 ± 1.08*	2.49 ± 0.99*
	Day-5	2.42 ± 1.25	3.05 ± 3.25	2.33 ± 2.26*	2.97 ± 1.38*
TGF-β1	Day-1	2.94 ± 1.42	3.67 ± 1.42	2.73 ± 0.76*	4.51 ± 1.23*
	Day-5	2.76 ± 1.18	3.16 ± 1.24	2.56 ± 0.96*	3.99 ± 1.04*

MMP-9: Matrix Metalloproteinase-9; NOX2: NADPH Oxidase 2; TGF-β1: Transforming Growth Factor Beta 1; SD: Standard Deviation. *p = < 0.05, analyzed with Mann-Whitney analysis

We also compared the standard laboratory test (hemoglobin, leukocyte, thrombocyte, blood urea nitrogen, creatinine serum, aspartate transaminase, and alanine transaminase between each group) in late PCI + OMT + colchicine, late PCI + OMT, No Revas + OMT + colchicine, and No Revas + OMT. There was no significant difference in each group. We also tested the troponin for all of the patients and all of them showed positive results. The data are shown in Table 3.

**Table 3:** Standard laboratory markers

Standard Biomarkers	Late PCI + Colchicine (n = 25)	Late PCI + Placebo (n = 24)	No Revas + Placebo (n = 31)	No Revas + Colchicine (n = 22)	P *Value*
	Mean ± SD				
Hemoglobin (Hb)	13.74 **±** 1.66	14.65 ± 2.07	13.5 **±** 1.81	14.4 ± 3.2	0.134
Leukocyte	13.70 ± 3.70	13.15 **±** 4.57	14.04 **±** 4.50	14.49 ± 5.1	0.178
Thrombocyte	263.04 ± 45	294.20 ± 37	297.30 ± 54	301.40 ± 67	0.243
BUN	19.20 ± 11.48	17.25 ± 8.1	15.67 ± 4.8	23.09 ± 17.8	0.192
Cr	1.26 ± 0.41	1.17 ± 0.30	1.57 ± 0.12	1.35 ± 0.95	0.332
AST	76.72 ± 14.4	70.95 ± 9.1	74.78 ± 15.9	78.09 ± 10.8	0.212
ALT	35 ± 8.7	40.78 ± 7.6	41.21 ± 7.5	49.58 ± 9.79	0.412

## Discussion

This study focuses on the investigation of colchicine and its benefit to reduce MMP-9, NOX-2, and TGF-β1 levels through ELISA test evaluation in stable STEMI patients. The demographic data of patients including age, gender, and risk factors such as hypertension, diabetes, dyslipidemia, smoker and ex-smoker, and also the large number of infarcts did not show the significant differences, so the results didn’t influence the distribution of patients in each group. The standard laboratory data like Hb, leukocyte, thrombocyte, BUN, Cr, AST, and ALT levels also did not show the significant differences between each group, so those standard markers didn’t influence the distribution and result in each group.

### Understanding cardiac remodeling

It is essential to understand the process of cardiac remodeling before interpreting the results of this study. Physiological cardiac remodeling and pathological cardiac remodeling are the two kinds of cardiac remodeling. Physiological cardiac remodeling occurs as a result of healthy exercise and endurance training, while pathological cardiac remodeling occurs due to chronic stresses such as hypertension, volume overload, neuroendocrine activation, and MI [18]. Heart failure (HF) progression is closely associated with pathological cardiac remodeling [9]. A series of unfavorable modifications, such as interstitial fibrosis, contractile failure, energy deficit, cardiomyocyte death, vascular dysfunction, and chamber dilatation typically follow the initial adaptive reaction of global or localized ventricular hypertrophy. These maladaptive changes are together known as adverse cardiac remodeling [19]. Additionally, extracellular matrix remodeling and heart dilation are important aspects of cardiac remodeling following MI. As the heart transitions from compensatory hypertrophy to dilated heart failure, these cellular and molecular modifications become more prominent, resulting in cardiomyocyte elongation, ECM remodeling, chamber dilation, and diminished systolic and/or diastolic function.

The extracellular matrix (ECM) is also altered in combination with cardiomyocyte death due to necrosis or apoptosis, which happens concurrently with these cellular and molecular modifications within the cardiomyocyte. The heart responds differently to stress and injury on a macroscopic level. After a myocardial infarction, the area of damage expands immediately, followed by regional dilatation and thinning. Similar variations in cardiomyocyte and microtubule cell structure are generally accompanied by cardiac remodeling as a whole. After a myocardial infarction, the length and width of cardiomyocytes may increase, while the thickness of the local ventricular wall may decrease. The apparent difference can be ascribed to alterations in wall structure, slippage between cardiomyocytes and the ECM, and a decline in cardiomyocyte quantity. After an acute MI, a complicated remodeling mechanism is initiated.

### MMP-9, NOX2, and TGF-β1 in cardiac remodeling

NOX2 plays an initial role in the development of cardiac remodeling. At the infarcted and penumbra location, oxidative stress will develop, activating NOX2 and AT1 receptors in cardiomyocytes and fibroblasts. NOX2 is triggered to generate nitric oxide and oxidative stress [20]. Persistent increases in mitochondrial oxygen radical synthesis can result in mitochondrial DNA damage and cellular damage due to an increase in oxygen radical production. This procedure may result in necrosis and apoptosis of cardiomyocytes. Apoptosis of cardiomyocytes does not induce an inflammatory response; rather, it induces a rapid phagocytic response by tissue macrophages [21]. CaMKII, a NOX2-dependent oxidation, causes the rise in MMP-9 production in cardiomyocytes, according to a research by He et al. MMP-9 and NOX2 may interact to promote the development of remodeling processes [22]. Chancey et al. found that MMP inhibitors may reduce myocardial remodeling by lowering LV hypertrophy and maintaining ventricular function [23]. MMPs contribute to cardiac remodeling by stimulating ECM protein production. Fibroblasts, myocytes, and endothelial cells create MMPs. MMPs control ECM turnover by denaturing and degrading fibrillar collagen [24]. Due to the rise in MMP-9 following MI, Halade et al. found that MMP-9 may serve as a proximal biomarker for myocardial remodeling [25].

The biomarker analysis showed that the level of TGF-β1 in OMT + Colchicine group is lower than OMT + Placebo group. An in vivo study using TGF-β1 inhibitors demonstrated the positive effects of TGF- β1 inhibition on maintaining heart function and minimizing cardiac fibrosis and remodeling [26]. TGF- β1 is activated by activation of NOX2 and MMP-9 via both SMAD-independent and SMAD-dependent mechanisms [27]. It begins 3–4 days after a myocardial infarction with the activation of TGF-1 and the entry of myofibroblasts at the infarct site, promoting the deposition of collagen fibers one week later. Activation of the angiotensin-1 (AT1) receptor and NOX2 in fibroblast and myocytes increases TGF-1 levels on a molecular level. There are two TGF-1 receptors in the cellular walls: TGF-1 RI and TGF-1 RII. These receptors activate smad effector proteins (smad 2/3, smad4, and smad 1/5). The activation of these cascades controls the deposition of fibrous tissue and the expression of ECM protein genes [20]. Replacement fibrosis will continue to accumulate throughout the following eight weeks. After scar tissue has restored the integrity of the infarcted heart, collagen turnover continues. In addition to occurring in non-infarcted myocardium, fibrous tissue contributes to the harmful structural remodeling of a failing ischemic heart [19].

### The role of colchicine in reducing cardiac remodeling process in no revas group

MMP-9, NOX2, and TGF-β1 analysis in OMT + Colchicine group is significantly different and lower than OMT + Placebo group: MMP9 (Day-1: p = 0.001; Day-5: p = 0.022), NOX2 (Day-1: p = 0.02; Day-5: p = 0.026), and TGF-β (Day-1: p = 0.00; Day-5: p = 0.00). The results may suggest the role of colchicine to regulate the biomarkers. Despite the molecular mechanisms, myocardial infarction also affects the microtubules of cardiomyocytes. During the transition from cardiac remodeling and cardiac hypertrophy to heart failure, several structural modifications in cardiomyocytes affect both systolic and diastolic activity. A doubled microtubule network has been identified as a possible reason for poor performance [28]. Microtubule proliferation considerably inhibits cardiomyocyte contraction, according to animal research on pressure overload-induced cardiomyopathy [29]. The relationship between microtubule growth and ROS has also been observed in a mouse experiment.

When there is an increase in ROS generation, mediated by NOX2, microtubule proliferation occurs in myocytes, and vice versa [30]. Hanania et al. found a link between microtubule and MMP-9 in an in vitro model [31]. Through the stimulation of many chemokines throughout the proliferation process, microtubules may function as a motor in MMP-9 synthesis. Prins et al. illustrate the effect of colchicine in inhibiting the proliferation of microtubules [32].

**Fig. 3:**
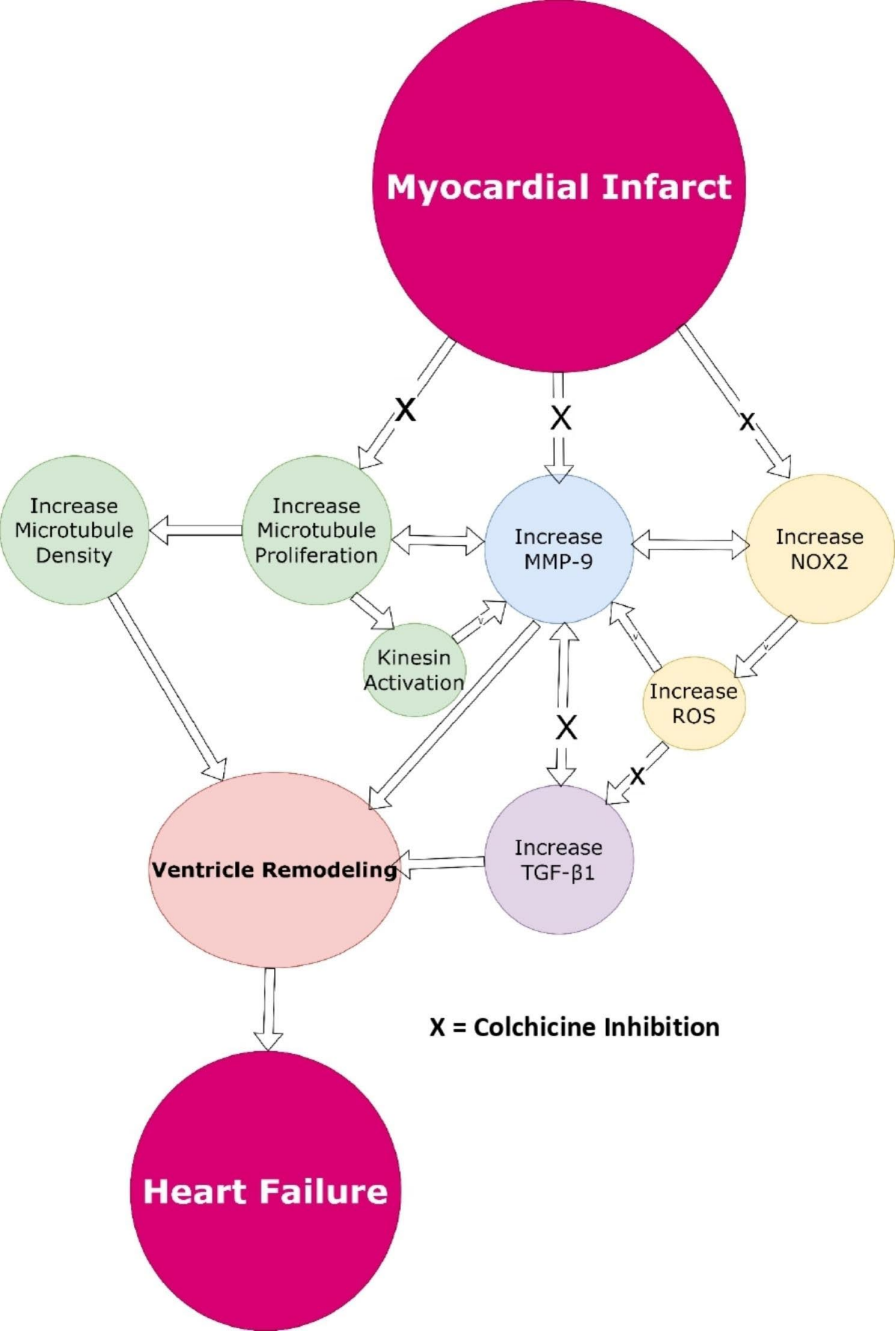
Colchicine mechanism to inhibit ventricle remodeling process

Colchicine improves right ventricular function and t-tubule architecture, whereas reducing microtubule density and junctophilin-2 expression. This study is supported by the molecular docking study by Suryono et al. that demonstrates colchicine may decrease several biomarkers like MMP-9, NOX2, and TGF-β1 [16]. Colchicine improves right ventricular function and t-tubule architecture, whereas reducing microtubule density and junctophilin-2 expression. According to the findings, colchicine has the capacity to limit the level of MMP-9, NOX2, and TGF-β1. This evidence showed colchicine’s efficacy in slowing the course of cardiac remodeling. Figure 3 showed the role of colchicine to reduce ventricle remodeling process.

### MMP-9, NOX2, and TGF-β1 in late PCI group

Based on the guidelines, it is certain that STEMI patients with an onset < 12 h will be treated with primary PCI or fibrinolytic [33]. However, the condition in several developing countries is different that the patients potentially come to the healthcare center when the onset is already > 12 h. A study conducted by Dharma et al. in 2016 stated that STEMI patients in Jakarta, the capital city of Indonesia, who got Primary PCI are just 35% and 2.2% got fibrinolytic, and the remaining 63% did not get revascularization [7]. That is why this study included patients with the onset of STEMI between 12 and 48 h.

Besides the results in the No Revas group already suggest the benefit of colchicine in reducing MMP-9, NOX2, and TGF-β1, the analysis in both late PCI groups is still not significant. If we compare the level of biomarkers in Fig. 2, the trends showed that the PCI + Colchicine group had lower MMP-9, NOX2, and TGF-β1 levels than the PCI + Placebo group. These results may happen because of the PCI intervention, since it may develop Ischemia/Reperfusion Injury (IRI) in the acute phase. IRI is a term used to describe the functional and structural abnormalities that occur when blood flow is restored after a period of ischemia. In addition to reversing ischemia, the restoration of blood flow can result in potentially highly damaging side effects, such as necrosis of irreversibly injured cells, significant cell swelling, and nonuniform flow restoration to all tissue regions [34].

IRI initially mobilizes neutrophils via chemotaxis and endothelial adhesion, CD4 + T cells, and circulating platelets in the vascular space. Neutrophils induce the generation of tissue-damaging reactive oxygen species (ROS), tumor necrosis factor-alpha (TNF-α), and local inflammatory mediators [35]. CD4 + T lymphocytes produce macrophage-stimulating factors, interferon-gamma, and TNF- α, which augment macrophage activation and cytokine release [35]. Furthermore, re-oxygenation increases the number of oxygen free radicals in the parenchymal, endothelial, and lymphocytic cells that infiltrate the lesion. The production of superoxide anions is due to the inadequate reduction of oxygen by damaged mitochondria and the action of neutrophils, endothelial cells, or parenchymal cells.

These processes lead to the formation of free radicals, which are unstable molecules that destabilize inorganic and organic substances and cause cell damage [36]. The activation of ROS and inflammation process in IRI increases the level of MMP-9, NOX2, and TGF-β1. The results may not be significant because the data extraction happens in Day-1 and Day-5 (acute phase). Therefore, further research is needed to confirm whether higher doses of colchicine may reduce MMP-9, NOX2, and TGF-β1 or not, since the results in the No Revas group (without IRI).

## Conclusion

Colchicine could significantly reduce MMP-9, NOX2, and TGF-β1 levels in stable STEMI patients. So that, colchicine could be a potential agent in STEMI patients and prevent cardiac remodeling events.

## Data Availability

The corresponding author will provide the dataset upon reasonable request, which was used to conduct the current work.
